# Asymmetric Dopaminergic Degeneration and Attentional Resources in Parkinson’s Disease

**DOI:** 10.3389/fnins.2018.00972

**Published:** 2018-12-17

**Authors:** Paola Ortelli, Davide Ferrazzoli, Marianna Zarucchi, Roberto Maestri, Giuseppe Frazzitta

**Affiliations:** ^1^Department of Parkinson’s Disease, Movement Disorders and Brain Injury Rehabilitation, Moriggia-Pelascini Hospital, Como, Italy; ^2^Istituti Clinici Scientifici Maugeri – Istituto di Ricovero e Cura a Carattere Scientifico, Biomedical Engineering Unit of Montescano Institute, Pavia, Italy

**Keywords:** Parkinson’s disease, attention, dopamine-related asymmetry, neuroplasticity, rehabilitation

## Abstract

**Background:** Attention is crucial to voluntary perform actions in Parkinson’s disease (PD), allowing patients to bypass the impaired habitual motor control. The asymmetrical degeneration of the dopaminergic system could affect the attentional functions.

**Objective:** To investigate the relationship between the asymmetric dopaminergic degeneration and the attentional resources in Parkinsonian patients with right-side (RPD) and left-side (LPD) motor symptoms predominance.

**Methods:** 50 RPD, 50 LPD, and 34 healthy controls underwent visual (V), auditory (A), and multiple choices (MC) reaction time (RTs) tasks. For PD patients, these tasks were performed before and after a 4-week intensive, motor-cognitive rehabilitation treatment (MIRT). The effectiveness of treatment was evaluated assessing Unified Parkinson’s disease Rating Scale (UPDRS) III and Timed-up and Go Test (TUG).

**Results:** RTs did not differ between PD patients and healthy controls. Before MIRT, no differences between LPD and RPD patients were observed in RTs (*p* = 0.20), UPDRS III (*p* = 0.60), and TUG (*p* = 0.38). No differences in dopaminergic medication were found between groups (*p* = 0.44 and *p* = 0.66 before and after MIRT, respectively). After MIRT, LPD patients showed a significant reduction in MC RTs (*p* = 0.05), V RTs (*p* = 0.02), and MC-V RTs. A significant association between changes in RTs and improvements in UPDRS III and TUG was observed in LPD patients.

**Conclusion:** attention does not differ among RPD patients, LPD patients and healthy controls. Only LPD patients improved their performances on attentional tasks after MIRT. We argue that the increased early susceptibility of the left nigrostriatal system to degeneration affects differently the cognitive modifiability and the neuroplastic potential. Our results could provide insight into new therapeutic approaches, highlighting the importance to design different treatments for RPD patients and LPD patients.

## Introduction

Parkinson’s disease (PD) is the second most common neurodegenerative disorder characterized by different motor and non-motor symptoms. An enigmatic feature of PD is the asymmetry of motor signs (Hoehn and Yahr, 1967; Hughes et al., 1992; Gelb et al., 1999), which persists throughout the spam of the disease progression and allows differentiating idiopathic PD from atypical Parkinsonian syndromes (Djaldetti et al., 2006).

The lateralization of motor symptoms is associated with a more severe contralateral degeneration of dopaminergic neurons (Haaxma et al., 2010), which is in turn responsible for a hypo-dopaminergic state in the striatum and frontal regions (Marie et al., 1995; Mattay et al., 2002; Cheesman et al., 2005). The correlation between dopamine innervation and expression of cognitive capacities (Nieoullon, 2002) indicates that dopamine-related asymmetry could impact on cognitive resources (Huber et al., 1989; St Clair et al., 1998; Tomer et al., 2007; Verreyt et al., 2011; Erro et al., 2013; Poletti et al., 2013; Pellicano et al., 2015). Different cross-sectional studies explored this topic both in drug naïve and medicated PD patients at different disease stages, yielding mixed results.

In a review of 36 published studies, Verreyt et al. (2011) concluded that PD patients with right-side motor symptoms predominance (RPD) mostly present problems in language and verbal memory tasks, whereas PD patients with left-side motor symptoms predominance (LPD) show impairments in visuospatial orienting, spatial attention and mental imagery (Verreyt et al., 2011).

Cognition is improved by specific motor trainings (David et al., 2015; Ferrazzoli et al., 2017), and it has been showed how the improvement in cognitive functioning may be considered as an index of neuroplasticity (Hötting and Röder, 2013).

Given these premises, it is conceivable that dopamine-related asymmetry could impact both on cognition and cognitive modifiability. The assessment of attention can be considered as a valid tool to explore these topics (Cohen, 1993).

We previously demonstrated that attention does not differ between healthy controls and PD patients (Ferrazzoli et al., 2017). In order to understand to what extent dopamine-related asymmetry affects attention and the cortical functioning, in this study we have examined specific attentional processes (indirectly related to the basal ganglia) (Posner and Petersen, 2012) in RPD patients, LPD patients and healthy controls, separately. A widespread method to measure attention is the reaction times (RTs) measurement (Jensen, 2006), which represents the elapsed time between a sensory stimulus presentation and the following behavioral response (Jensen, 2006). Using auditory (A) and visual (V) RTs we have evaluated the auditory and the visual attention (i.e., the sensory dimension of attention), whereas using V and multiple choices (MC) RTs we have evaluated the alertness and the focused and sustained attention (i.e., the functional dimension of attention).

Data from animal models and humans support the role of exercise in restoring plasticity at the level of both motor and cognitive circuitries in PD (Fisher et al., 2004; Petzinger et al., 2013; Frazzitta et al., 2014; da Silva et al., 2016; Fontanesi et al., 2016; Hirsch et al., 2016; Chen et al., 2017).

Rehabilitation in PD is aimed to re-learn and correctly execute the lost habitual motor behaviors (Ferrazzoli et al., 2017; Ferrazzoli et al., 2018a). The executive resources are exploitable for these purposes (David et al., 2015; Ferrazzoli et al., 2017; Ferrazzoli et al., 2018a), and it has been found that the executive component of attention (i.e., the focused and sustained attention) is modifiable in parkinsonian patients who undergo a high demanding motor-cognitive and goal-based trainings (Morris, 2006; Morris et al., 2008; Nieuwboer et al., 2009; Morris et al., 2010; David et al., 2015; Ferrazzoli et al., 2017; Ferrazzoli et al., 2018a).

To evaluate whether dopamine-related asymmetry affects the cognitive modifiability and the benefits from rehabilitation, RPD and LPD patients underwent the same clinical-functional assessment and RTs tasks also after a 4-week multidisciplinary, motor-cognitive intensive rehabilitation treatment (MIRT).

## Materials and Methods

### Study Population

Between January and August 2017, we enrolled at the Department of Parkinson’s disease, Movement Disorders and Brain Injury Rehabilitation (“Moriggia-Pelascini” Hospital, Gravedona ed Uniti, Como, Italy), 50 RPD and 50 LPD right-handed patients, hospitalized for a 4-week MIRT (Frazzitta et al., 2012, Frazzitta et al., 2015b; Ferrazzoli et al., 2018b). 34 healthy right-handed subjects served as controls at baseline. Parkinsonian patients were diagnosed according to the UK Brain Bank criteria (Hughes et al., 1992) and were evaluated by a neurologist with experience in movement disorders.

The inclusion criteria were: (i) stage 2.5–3 according to the Hoehn and Yahr scale (H&Y); (ii) stable pharmacological treatment for the last 6 weeks before the enrolment and during the hospitalization; (iii) Mini Mental State Examination (MMSE) ≥ 24 (Folstein et al., 1975); (iv) no evidences of dysexecutive syndrome (Godefroy et al., 2010); (v) DaT SPECT scans with 123-ioflupane (DaT-SCAN) reporting reduced ligand uptake contralateral to the clinically more affected side (Bajaj et al., 2013).

Exclusion criteria were: (i) any focal brain lesion detected in brain imaging studies (CT or MRI) performed in the previous 12 months; (ii) drug-induced dyskinesias; (iii) disturbing resting and/or action tremor, corresponding to scores ≥ 2 in the specific tremor items of Unified Parkinson’s Disease Rating Scale (UPDRS) III; (iv) behavioral disturbances (evaluated with Neuropsychiatric Inventory); (v) visual and auditory dysfunctions according to the general clinical evaluation and medical history; (vi) equivocal report about the side of disease onset or bilateral motor involvement.

Patients’ lateralization of the disease was based on the initial neurological examination and the reports regarding the side and presentation of motor symptoms. This was accomplished combining history data with the UPDRS III scores in terms of laterality (Plotnik et al., 2005; Ricciardi et al., 2015).

The study design and protocol were approved by the local Ethics Committee (“Comitato Etico Interaziendale delle Province di Lecco, Como e Sondrio”) and were in accordance with the code of Ethics of the World Medical Association (Declaration of Helsinki, 1967). A complete explanation of the study protocol was provided and written informed consent was obtained from all participants before their participation in the study. This trial was registered on ClinicalTrials.gov website (NCT03476668).

### Neuropsychological Assessment

Neuropsychological assessment included the MMSE (Folstein et al., 1975; Magni et al., 1996) and the frontal assessment battery (FAB) (Dubois et al., 2000; Appollonio et al., 2005).

### Attentional RTs Tasks

**FIGURE 1:**
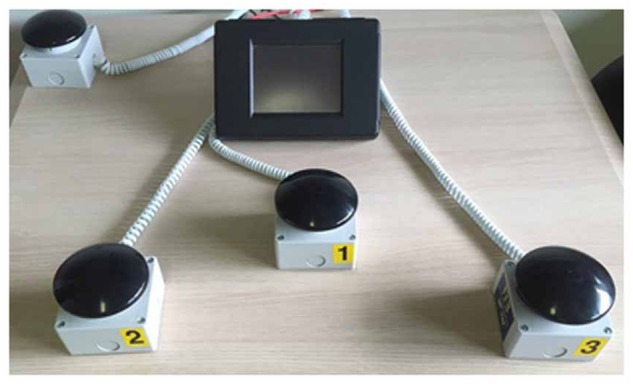
Device used to assess attention. System used to assess attention by the evaluation of the performance in a computer-controlled reaction times paradigm (ITB Sport Reflection, F.M. Automazione S.r.l., Brescia, Italia).

Attention was assessed by the evaluation of the performance in a randomized computer-controlled RTs paradigm (ITB Sport Reflection, F.M. Automazione S.r.l., Brescia, Italia; see Figure 1). For PD patients, V RTs, A RTs, and MC RTs were assessed at 9 AM, during the medication “on” state, at the enrolment and at the end of MIRT. Healthy controls performed the same RTs tasks at the enrolment, in the morning. These subjects served only for comparison to evaluate the attentional resources in PD. Therefore, healthy controls did not undergo any physical treatment and performed the attentional tasks only once, at baseline. V RTs, A RTs, and MC RTs were assessed in a randomized order, both for PD patients and healthy controls. The entire test session lasted 45 min.

Subjects were asked to seat directly in front of a 5.7″ diagonal monitor (display resolution 320 × RGB × 240; Pixel Pitch 0.36 H × 0.36 V; active area 115.2 W × 86.4 H; outline dimension 144.0 W × 140.6 H × 12.8 T without FPCB tail; color garmut NTSC 58%) at a distance that was comfortable to them. The investigators read the instructions before the starting of experimental tasks. For each task, subjects performed one training section in order to become confident with the experiment and avoid the bias related to the learning effect of test-retest. Subjects were instructed to place their preferred hand on a table, always at the same distance from the response buttons, in a specific position indicated with a black line. Subjects had to look at the screen and press the response key (response button) when a target-stimulus appeared using their preferred hand. Between the stimuli, the subject had to remain with the hand at rest on the table.

#### Visual Reaction Times Task

**FIGURE 2:**
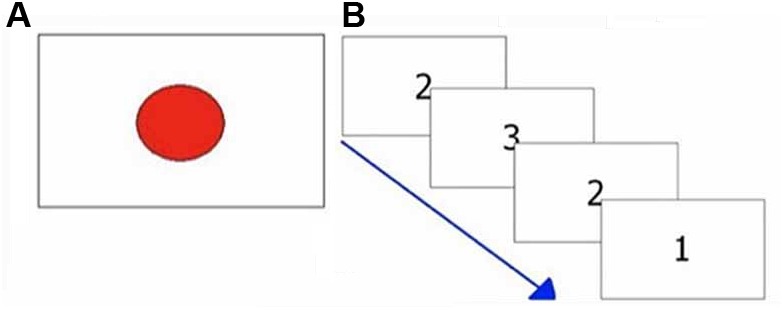
Exemplifications of attentional tasks. **(A)** Visual reaction times task; **(B)** Multiple choices reaction times task.

The task consisted of 40 trials. In each single trial, the subjects had to press as quickly as possible a response button at the appearance of a red circle presented at irregular intervals (1–3 s) in the center of the screen (see Figure 2A). This target disappeared after the subject’s response. The elapsed times between the appearance of circles and the subjects’ responses were recorded. Response times shorter than 250 ms and longer than 1000 ms were deemed to be outliers and were excluded from analysis. The number of RTs excluded from the analysis was recorded. The median value was taken as representative of the central tendency of each subject (Ratcliff, 1993; Ratcliff and Van Dongen, 2011).

#### Auditory Reaction Times Task

The task consisted of 40 trials. In each single trial the subjects had to press as quickly as possible a response button when hearing an acoustic stimulus (intensity of 94 dbA). The stimuli were presented at irregular intervals (1–3 s) and ended after the subject’s response. The elapsed times between the presentation of the acoustic stimuli and the subjects’ responses were recorded. Response times shorter than 250 ms and longer than 1000 ms were deemed to be outliers and were excluded from analysis. The number of RTs excluded from the analysis was recorded. The median value was taken as representative of the central tendency of each subject.

#### Multiple Choices Reaction Times Task

The task consisted of 40 trials. A number (1, 2, 3) was randomly presented in the center of the screen. Each number was associated to a different response button (see Figure 1). Subjects had to press the button associated with the number shown on the screen (see Figure 2B), as quickly as possible.

The accuracy of responses was evaluated by counting the number of errors. The elapsed times between the presentation of the stimuli and the subjects’ responses were recorded. RTs shorter than 250 ms and longer than 2000 ms were deemed to be outliers and were excluded. The number of RTs excluded from the analysis was recorded. The median value of valid response times was taken as representative of the central tendency of each subject.

#### Subtraction Method for Reaction Times

We subtracted V RTs from MC RTs for obtaining an estimate of the time required for the executive attentional processes (subtractive RT) *per se* without considering the sensory component.

### Rehabilitation Treatment

MIRT is a multidisciplinary, aerobic, motor-cognitive, intensive and goal-based rehabilitation treatment, specifically designed for PD patients (Frazzitta et al., 2012; Frazzitta et al., 2015b; Ferrazzoli et al., 2018b). Aim of the treatment is to re-learn the dysfunctional movements resulting from the disease using explicit and implicit learning strategies. It consists of a 4-week program in a hospital setting, composed of four daily rehabilitative sessions for 5 days and 1 of physical exercise on the sixth day. The duration of each session, including recovery periods, is about 1 h:

- The first session consists of a one-to-one treatment with a physical therapist tailored on patient’s individual motor and cognitive abilities. It comprises cardiovascular warm-up activities, active and passive exercises to improve the joints range of motion, stretching of the abdominal muscles, strengthening of paravertebral muscles, postural changes and exercises for balance and postural control.

- The second session is based on the use of various devices to improve gait, balance, endurance and motor control. The devices adopted are i) a stabilometric platform with biofeedback→ Subjects underwent a 15 min balance training, using a stabilometric platform (Prokin 254, TecnoBody S.r.l, Dalmine 24044, Bergamo, Italy) in monopodal and bipodal standing for six days per week, for 4 weeks. Using biofeedback, patients were asked a) to maintain a cursor sensitive to the displacement of the center of gravity within a target located in the center of the screen, and b) to reach scattered circles by following tracks of different shapes and lengths; ii) a treadmill plus (treadmill training with visual cues and auditory feedback)→ We used a motorized medical treadmill (Gait Trainer 3 Biodex, Biodex Medical System – 20 Ramsay Road, Shirley, New York, United States) for gait training. All patients underwent a 30 min treadmill training per day (divided in two 15 min sessions), 6 days a week, for 4 weeks, with the supervision of a physiotherapist with expertise in movement disorders rehabilitation. The belt speed was initially set at 1.5 km/h and it was progressively increased to a maximum of 3.5 km/h, depending on the physical ability of each patient. The visual cue consisted of a target defined by two horizontal lines, displayed on a screen. The space between the two lines was calculated for each patient according to gender, height, age and normative data. The right and left patients’ footprints were shown alternatively on the screen: when they fell within the space delimited by the two lines, a “well done” message appeared; otherwise, patients were invited to take longer or shorter steps in order to adapt the stride length to the set target. The auditory feedback consisted of musical beats with a frequency of 0.5 c/s and synchronized with the visual cues; iii) a crossover (a type of cross-trainer designed for cardiovascular exercises)→ Each patient underwent a 4-week cycle of crossover training (Technogym^®^) for 6 days per week; iv) a cycloergometer with feedback.

- The third session consists of occupational therapy aimed to improve the autonomy in everyday activities. The session focuses on hand dexterity, writing and activities of daily living.

- The fourth session includes one hour of speech therapy aimed to treat the hypokinetic dysarthria. During this session patients underwent breathing exercises to relax and alleviate the pressure of speech, facial exercises to improve the range of facial expressions and mouth motion, and exercises to improve vocalization, articulation and speech prosody.

On the sixth day the patients are trained only with devices for one hour.

The rehabilitation program entails also a psychoeducational therapy with neuropsychologists (1-hour treatment *per* week, for 4 weeks) and could also include robotic-assisted walking training for complex gait disorders (three 1 h sessions *per* week, for 4 weeks) and virtual-reality training (three 1 h sessions *per* week, for 4 weeks).

During all the activities, the heart rate reserve is kept between 70 and 80%.

A weekly team meeting defines the rehabilitation program for each patient and assesses its benefits during the course of the hospitalization.

### Clinical Evaluation and Outcome Measures

A neurologist with experience in movement disorders examined the patients in the morning, 1 h after they had taken the first dopaminergic drug dose, in medication “on” state, both at the beginning and at the end of MIRT. Levodopa equivalent dose (LED) was calculated for all the dopamine replacement therapies that patients were taking, using a standardized formula (Tomlinson et al., 2010). Healthy controls were evaluated at the same time of the day. All subjects were tested in a laboratory setting, with constant artificial lighting condition and in absence of auditory interferences. UPDRS III and the Timed Up and Go test (TUG) were assessed in order to investigate the clinical and motor-functional effectiveness of MIRT.

### Statistical Analysis

The central tendency and dispersion of continuous variables were reported as mean ± SD. Descriptive statistics for categorical variables were reported as N (percent frequency). The normality of all variables was assessed by Shapiro–Wilk statistic, supported by visual inspection. Since several variables did not satisfy the normality assumption, non-parametric tests were used. Between-group comparisons (controls, RPD patients and LPD patients) of baseline continuous demographic and cognitive variables and of values of RTs were carried out by the Kruskal-Wallis test, followed by *post hoc* analysis (Tukey–Kramer adjustment) to compare pairs of groups. Between-group (RPD vs LPD patients) comparison of clinical and functional variables was carried out by the Mann-Whitney U-test. To assess the effect of MIRT on clinical outcomes and RTs, the difference between the discharge and admission values (Deltas) were computed and the null hypothesis that the Deltas were from a distribution with median zero was tested by the Wilcoxon signed rank test. To test the hypothesis that MIRT could affect differently the rehabilitation outcome in terms of RTs depending on the side of motor symptoms predominance, the difference between the discharge and admission values were compared between-group (RPD vs LPD patients), thus testing for the interaction between treatment and side of motor symptoms predominance. The association between variables was assessed by Spearman rank correlation coefficient. All statistical tests were two-tailed and statistical significance was set at *p* < 0.05. All analyses were carried out using the SAS/STAT statistical package, release 9.4 (SAS Institute Inc., Cary, NC, United States).

## Results

The rate of errors in accomplishing the MC RTs trials was very low for all controls and patients, regardless of the side of motor symptoms predominance, ranging from 0 to 3 out of the 40 trials. Globally, 94% of evaluations were without errors.

Baseline values of demographic and cognitive variables for healthy controls and PD patients are reported in Table 1. *Post hoc* analysis revealed significant differences only in MMSE (LPD vs. RPD, *p* = 0.019, LPD vs. Controls, *p* = 0.002) and in FAB (LPD vs. Controls, *p* = 0.029). Baseline RTs for healthy controls and PD patients are reported in Table 2. No significant differences were observed between healthy controls and RPD and LPD patients.

**Table 1:** Baseline values of demographic and cognitive variables for controls and PD patients grouped according to the side of motor symptoms predominance.

Variable	Controls	RPD	LPD	*p*	Chi square
Gender (% of males)	68	41	50	0.063	5.4
Age (years)	65.4 ± 7.1	66.9 ± 9.2	64.0 ± 9.8	0.32	2.26
Education (years)	10.4 ± 4.3	10.3 ± 4.0	10.7 ± 5.0	0.98	0.05
MMSE	28.7 ± 1.3	27.2 ± 1.7^†^	27.4 ± 2.3^‡^	0.001	13.02
FAB	3.0 ± 0.9	1.9 ± 1.7^∧^	2.2 ± 1.7	0.039	6.51


*Reported p-values are from the Kruskal–Wallis test (between-group comparison, Controls, RPD and LPD) or from the chi-squared test for gender. Post hoc comparisons (Tukey–Kramer): ^‡^*p* = 0.019 LPD vs. Controls; ^†^p = 0.002 RPD vs. Controls; ˆp = 0.029 RPD vs. Controls.*

*RPD, patients with right-side motor symptoms predominance; LPD, patients with left-side motor symptoms predominance; MMSE, Mini mental state examination; FAB, Frontal assessment battery.*

**Table 2:** Baseline values reaction times for controls and PD patients grouped according to the side of motor symptoms predominance.

Variable	Controls	RPD	LPD	*p*	Chi square
MC RTs (ms)	926.63 ± 170.44	972.98 ± 183.91	965.92 ± 183.48	0.49	1.43
V RTs (ms)	342.10 ± 82.94	323.04 ± 60.29	348.14 ± 89.37	0.40	1.85
A RTs (ms)	290.46 ± 87.64	292.99 ± 70.47	307.97 ± 113.58	0.77	0.53
Subtractive RTs (ms)	584.53 ± 164.81	649.60 ± 158.23	617.79 ± 156.70	0.22	3.04


*Reported p-values are from the Kruskal–Wallis test (between-group comparison, Controls, RPD and LPD).*

*RPD, patients with right-side motor symptoms predominance; LPD, patients with left-side motor symptoms predominance; MC, Multiple choices; V, Visual; A, Auditory; RTs, Reaction times; ms, milliseconds.*

Table 3 reports baseline clinical and functional data for PD patients as a whole and stratified according to the side of motor symptoms predominance.

**Table 3:** Baseline clinical and functional data for patients as a whole and stratified according to the side of motor symptoms predominance.

Variable	All patients	RPD	LPD	*p*	*z* statistic
Disease duration (years)	10.6 ± 5.3	11.5 ± 5.6	9.6 ± 4.8	0.24	-1.17
H&Y	2.6 ± 0.5	2.6 ± 0.5	2.5 ± 0.5	0.53	-0.62
LED (mgeq/die)	713.9 ± 318.9	738.9 ± 374.7	679.5 ± 222.0	0.45	-0.76
UPDRS III	18.4 ± 5.3	17.9 ± 5.3	19.0 ± 5.3	0.58	0.55
TUG (s)	11.4 ± 5.5	11.7 ± 6.3	11.0 ± 4.2	0.78	-0.28


*Reported p-values are from the Mann-Whitney U-test (between-group comparison, RPD vs. LPD).*

*RPD, patients with right-side motor symptoms predominance; LPD, patients with left-side motor symptoms predominance; H&Y, Hoehn and Yahr stage; LED, Levodopa equivalent dosage; UPDRS, Unified Parkinson’s disease Rating Scale; TUG, Timed Up and Go test; mgeq, milligram equivalent; s, seconds.*

The changes in LED, UPDRS III, and TUG, after rehabilitation, are shown in Table 4. After MIRT, LED was significantly reduced and UPDRS III and TUG improved significantly in the overall population, as well as in both subgroups of RPD and LPD patients (*p* < 0.001 all). No difference in the strength of the improvement between RPD and LPD patients was observed. Changes in RTs are reported in Table 5, together with values at discharge. Considering all patients, a significant improvement was observed in MC RTs, V RTs and subtractive RTs (*p* < 0.05 all) but not in A RTs.

**Table 4:** Changes (Δ = values at discharge – values at admission) in LED, UPDRS III, and TUG for patients as a whole and stratified according to the side of motor symptoms predominance.

Variable	All patients	RPD	LPD	*P*	z statistic
Δ LED (mgeq/die)	-65.5 ± 123.8^‡^	-63.7 ± 123.3^‡^	-68.1 ± 126.5^†^	0.66	0.43
Δ UPDRS tot	-12.7 ± 4.5^‡^	-12.5 ± 4.4^‡^	-12.9 ± 4.7^‡^	0.76	-0.31
Δ UPDRS III	-5.4 ± 3.2^‡^	-5.2 ± 3.5^‡^	-5.727 ± 2.7^‡^	0.80	0.26
Δ TUG (s)	-2.63 ± 3.06^‡^	-2.98 ± 3.59^‡^	-2.14 ± 2.0^‡^	0.39	0.93


*Reported p-values are from the Mann-Whitney U-test (between-group comparison, RPD vs. LPD).*

*^‡^p < 0.001; ^†^p < 0.01; Wilcoxon signed rank test testing H0: Deltas are from a distribution with median = 0.*

*RPD, patients with right-side motor symptoms predominance; LPD, patients with left-side motor symptoms predominance; LED, Levodopa equivalent dosage; UPDRS, Unified Parkinson’s disease Rating Scale; TUG, Timed Up and Go test; mgeq, milligram equivalent; s, seconds.*

**Table 5:** Values at discharge and changes (Δ = values at discharge – values at admission) in reaction times for patients as a whole and stratified according to the side of motor symptoms predominance.

	All patients		RPD		LPD			
					
Variable	Discharge	Δ	Discharge	Δ	Discharge	Δ	p	z stat
MC RTs (ms)	951 ± 155	-34 ± 121ˆ	921 ± 193	-26 ± 131	951 ± 155	-44 ± 108†	0.09	-1.68
V RTs (ms)	321 ± 57	-9 ± 46ˆ	330 ± 96	-2 ± 47	321 ± 57	-18 ± 44ˆ	0.23	-1.21
A RTs (ms)	290 ± 80	-7 ± 75	297 ± 122	-3 ± 60	290 ± 80	-11 ± 93	0.78	-0.27
Subtractive RTs (ms)	630 ± 130	-24 ± 109ˆ	591 ± 156	-22 ± 116	630 ± 130	-27 ± 101ˆ	0.41	-0.82


*Reported p-values are from the Mann-Whitney U-test (between-group comparison of changes, RPD vs. LPD). ^‡^p < 0.001; ^†^p < 0.01; ˆp < 0.05; Wilcoxon signed rank test testing H0: Deltas are from a distribution with median = 0.*

*RPD, patients with right-side motor symptoms predominance; LPD, patients with left-side motor symptoms predominance; MC, Multiple choices; V, Visual; A, Auditory; RTs, Reaction times; ms, milliseconds.*

Stratifying by the side of motor symptoms predominance, it can be seen that global improvement in MC RTs, V RTs, and subtractive RTs was essentially due to LPD patients since RPD patients did not improve in RTs tasks.

**FIGURE 3:**
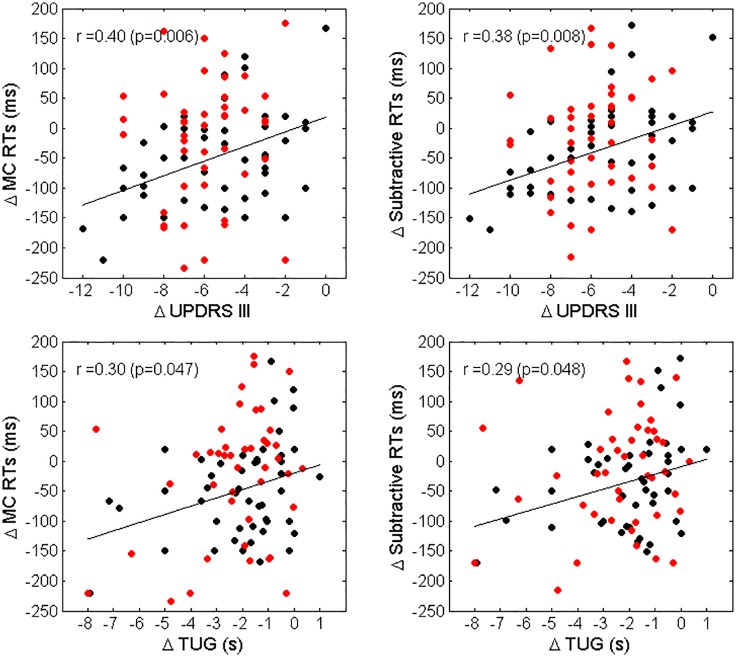
Scatterplot representation of Δ MC RTs vs. Δ UPDRS III (top left panel), Δ Subtractive RTs vs. Δ UPDRS III (top right panel), Δ MC RTs vs. Δ TUG (bottom left panel) and Δ Subtractive RTs vs. Δ TUG (bottom right panel). Black dots represent LPD patients, red dots RPD patients. The regression lines and the values of Spearman correlation coefficient r and significance for LPD patients only, are also reported.

A significant association between improvements in MC RTs and subtractive RTs and improvements in UPDRS III and TUG was observed for LPD patients only (*r* = 0.40, *p* = 0.006, and *r* = 0.30, *p* = 0.047 for the relationship between changes in MC RTs vs. changes in UPDRS III and TUG, respectively; *r* = 0.38, *p* = 0.009 and *r* = 0.29, *p* = 0.048 for the association between changes in subtractive RTs vs changes in UPDRS III and TUG, respectively). A scatterplot representation of these relationships is given in Figure 3. Finally, no association between changes in RTs and changes in LED was observed.

## Discussion

The main finding of this study is that the attentional resources do not differ among RPD patients, LPD patients and healthy controls. Instead, dopamine-related asymmetry seems to play a role on the modifiability of the attentional resources, as we found that a motor-cognitive, intensive and goal-based rehabilitation treatment is effective in improving attention in LPD patients, but none in RPD patients.

To the best of our knowledge this is the first study that analyzed the relationship between the asymmetry of neurological signs and attention by using RTs in PD patients. Specifically, we evaluated two different dimensions of attention: the sensory dimension by studying auditory and visual attention, and the functional dimension by studying alertness and focused and sustained attention.

About the sensory dimension, we observed that A RTs were 20–50 ms faster than V RTs. This finding is in line with previous data (Thaut et al., 1999; Nombela et al., 2013), showing that auditory stimuli are faster than visual ones. This is due to the reticulo-spinal connections that allow auditory stimuli to exert a more direct influence on the spinal-motor neurons pathway, reducing the time needed to generate a movement after a presentation of a sudden sound (Rossignol and Jones, 1976; Lee et al., 1996).

These data, together with the evidence that patients with PD show an impairment in visuo-spatial functions (Levin et al., 1991), suggest that the auditory cueing stimulation could be much more effective than the visual one.

About the functional dimension, the lack of differences in A RTs, MC RTs, and subtractive RTs measurement between patients with PD and healthy controls is not surprising, for two reasons: i) several neurotransmitters, other than dopamine, are involved in the attentional processes (Vossel et al., 2014), ii) patients were tested in medication “on” state. The latter is a relevant point, given the efficacy of dopaminergic drugs in improving such executive functions (Cools et al., 2001). These theoretical assumptions and the clinical-neuropsychological evidence explain why bradykinesia and bradyphrenia do not necessarily represent concurrent conditions, because while bradykinesia is something distinctive of PD, bradyphrenia occurs in certain cognitive or pharmacological states or in relation with the kind of task demand (Ferrazzoli et al., 2017).

Our findings indicate that the alertness and the executive component of attention are preserved in the early-medium PD stages in medication “on” state, both in LPD and RPD patients, without differences between groups. Attention is directly involved in the control of motor behavior (Logue and Gould, 2014) and it has been considered to be largely lateralized to the prefrontal and fronto-parietal regions of the right hemisphere (Corbetta and Shulman, 2002). However, our data suggest that the attentional functions are not strictly lateralized in the right hemisphere and that also left-hemispheric areas and circuits are involved in these processes (DiQuattro and Geng, 2011). Coherently, the only study (Bentin et al., 1981) that investigated the sustained attention in asymmetric PD subjects did not reveal differences between RPD and LPD patients (Bentin et al., 1981; Verreyt et al., 2011). Moreover, other authors were unable to report differences between RPD and LPD patients in tasks exploring attention in all its different components (Viitanen et al., 1994; Verreyt et al., 2011). The implications from these data are relevant, since alertness and focused and sustained attention are the basic requirement for the effectiveness of trainings based on the use of cueing techniques for patients with PD (Morris, 2006; Morris et al., 2008, 2010; Ferrazzoli et al., 2017; Ferrazzoli et al., 2018a).

The changes in RTs following MIRT were different in the two groups: while RPD patients did not showed modifications, LPD patients showed a significant reduction in V RTs, MC RTs and subtractive RTs, but none in A RTs. These results testify that the visuo-spatial component of alertness and the executive component of attention, but nor the activation timing of the motor-auditory loops, are modifiable after a motor-cognitive, intensive rehabilitation. LED was comparable in both groups at the end of treatment, indicating that the changes we found in RTs in LPD patients were due to rehabilitation and nor to dopaminergic drugs or to a generic improvement of the speed of information processing (Bowling and Mackenzie, 1996).

Despite the differences in RTs changes following MIRT, the improvement in motor-functional measures (UPDRS III and TUG) was similar in RPD and LPD patients. Nevertheless, a significant association between reductions in subtractive RTs and V RTs and improvements in UPDRS III and TUG was observed only in LPD patients, suggesting that the mechanisms that drive the motor behaviours and the (re-) learning of habitual skills are different in RPD and LPD patients.

A different neuroplastic potential between RPD and LPD patients could explain these differences in the modifiability of the attentional functions following rehabilitation. Many data support this hypothesis: first of all, a left hemispheric predominance of nigrostriatal dysfunction has been described in PD (Scherfler et al., 2012). In a large-scale prospective study Baumann et al. (2014) demonstrated that RPD patients show a more rapid motor symptoms progression in comparison to LPD patients (Baumann et al., 2014). Moreover, decreased muscle strength on both sides of the body compared to healthy controls was found in RPD patients, whereas no such differences were demonstrated in LPD patients (Frazzitta et al., 2015a). Heinrichs-Graham et al. (2017) demonstrated that LPD patients had stronger beta-suppression during movement compared to RPD patients and suggested that LPD patients present a more physiologic oscillatory pattern in comparison to RPD patients (Heinrichs-Graham et al., 2017). Further, early in the PD course, pathological cortical changes seem to mainly involve the left hemisphere (Claassen et al., 2016). Consistently with these findings, we found that MMSE and FAB scores were worst in RPD patients than in LPD patients, despite both of them fell within the range of normal values. The reason why the left hemisphere in PD exhibits an early susceptibility to degeneration is unknown and many hypotheses have been proposed, including an increased metabolic demand, increased oxidative stress and increased neurotoxicity with greater dopaminergic deficits (van der Hoorn et al., 2012). Although the left hemispheric predominance of nigro-striatal dysfunction has been found to be independent from handedness or the side of motor symptoms onset (Scherfler et al., 2012), it is unquestionable that RPD patients present a more left-lateralized nigro-striatal damage. Therefore, it is conceivable that patients predominantly affected in the right side, and suffering from left hemisphere pathology, present along the disease course a trend toward a more severe cognitive impairment, especially for attention and executive domains. Consequently, the non-modifiability of RTs in RPD patients following MIRT could be the expression of their reduced cognitive potentialities.

## Study Limitations

There are some limitations to this study that have to be acknowledged. First, we did not perform any semiquantitative analysis on DaT-SCAN SPECT imaging to establish the degree of asymmetry of the ligand uptake. Second, we did not perform a detailed neuropsychological assessment. This would allow us to better evaluate the relation between cognition and attentional performances. We did not collect follow-up data neither in LPD patients nor in RPD patients. Therefore, we cannot say how long the improvements we found last for and whether these changes may have been related or not to neuroplastic effects. We included PD patients with a mean H&Y stage of 2.5–3 in order to observe to what extent the asymmetric dopaminergic degeneration affects cognition in PD, mostly when subjects are treated with dopamine replacement therapy as it happens in the real-life clinical context. We did not include de novo drug-naive patients and this could be considered another limitation of this study. Finally, we did not evaluate the effect of a no goal-based treatment on RTs, so that we do not know whether the changes in attention following MIRT were due to cognitive engagement or had been related to a non-specific effect of exercise. Further studies are needed to address these questions.

## Conclusion

We found that the side of motor symptoms predominance does not affect the attentional resources in PD. A motor-cognitive, intensive and goal-based rehabilitation modifies attention in LPD but none in RPD patients. These differences in the modifiability of the attentional functions following rehabilitation could be related to differences in neuroplasticity between RPD and LPD patients. Our results could provide some insights into new therapeutic approaches, highlighting the importance to design different treatments for RPD patients and LPD patients.

## Author Contributions

PO and DF designed the research, wrote the text, provided substantial contributions to discussion of the content, and edited the manuscript before submission. PO and MZ researched data for the paper and performed the experiments. RM analyzed the data, did the statistical analysis and generated tables and figures. GF wrote the text, provided substantial contributions to discussion of the content and did a critical revision.

## Conflict of Interest Statement

The authors declare that the research was conducted in the absence of any commercial or financial relationships that could be construed as a potential conflict of interest.
